# Giant Cell Arteritis Presenting as Choroidal Infarction

**DOI:** 10.1155/2013/597398

**Published:** 2013-03-18

**Authors:** Nikolaos Kopsachilis, Maria Pefkianaki, Anca Marinescu, Sobha Sivaprasad

**Affiliations:** Moorfields Eye Hospital, 162 City Road, London EC1V 2PD, UK

## Abstract

*Background*. Giant cell arteritis (GCA) is a systemic granulomatous vasculitis that affects large- and medium-sized arteries of the head and neck. Ocular manifestations of GCA usually are anterior ischaemic optic neuropathy (AION) or retinal vessel occlusion. *Case Report*. We report an interesting case of a 70-year-old man who presented with sudden vision loss and choroidal infarction in his left eye. Thorough clinical and paraclinical evaluation revealed an underlying GCA, the treatment of which prevented further vision loss and systemic complications. *Conclusion*. This is an unusual presentation of choroidal infarction associated with CGA and emphasizes the need of thorough systemic evaluation in patients with choroidal infarction.

## 1. Introduction

Giant cell arteritis (GCA) is a systemic granulomatous vasculitis that affects large- and medium-sized arteries of the head and neck. Clinical features may include headache, jaw claudication, fever, visual disturbance, and raised serologic inflammatory markers such as the erythrocyte sedimentation rate (ESR) or C-reactive protein (CRP).

Ocular manifestations of GCA are anterior ischaemic optic neuropathy (AION) or retinal vessel occlusion. Rarely, it can cause tonic pupil. Temporal artery biopsy is widely accepted as the gold standard for diagnosis. In the absence of typical signs and symptoms, diagnosis becomes more difficult, thus increasing the risk of severe complications [1].

We report the first case of peripheral and central choroidal infarction associated with GCA.

## 2. Case Report

A 70-year-old man was referred to our clinic with sudden vision deterioration in his left eye since 2 days. He had a positive history of arterial hypertension, diabetes mellitus, and asthma. Visual acuity (VA) was 6/6 in the right eye and 3/60 in the left eye. Intraocular pressures were 14 mmHg both sides. Slit lamp examination showed a deep anterior chamber in both eyes with no signs of inflammation.

**Figure 1:**
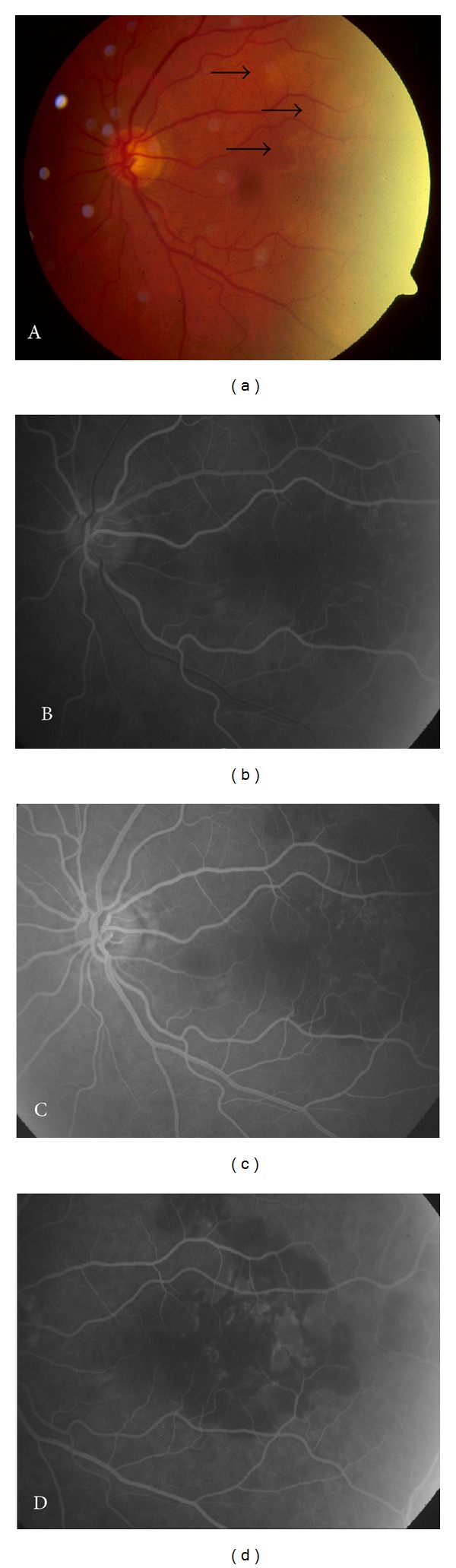
Fundus photograph of a 70-year-old patient with sudden vision deterioration in the left eye showing a large whitish area in the temporal retina. Although the photograph appears overexposed, black arrows indicate the whitish area of interest (a). Fluorescein angiogram of the left eye showed blockage corresponding to the whitish areas confirming the diagnosis of choroidal infarction (b), (c), and (d).

Fundoscopy of the left eye revealed a large whitish area in the temporal retina (Figure 1(a)). The right funduscopy findings were unremarkable. Fluorescein angiogram of the left eye showed blockage corresponding to the whitish areas without leakage confirming the diagnosis of choroidal infarction (Figures 1(b), 1(c), and 1(d)).

Full serology was investigated. Full blood count and carotid doppler was normal. Chest X ray was unremarkable. ESR was found to be 168 mm/hr, and CRP was 16.5 mg/dL. Based on the clinical and laboratory findings a biopsy of the temporal artery was arranged. Histopathologic examination of the specimens disclosed a mixed inflammatory cell infiltrate in the tunica media with more than one multinucleate giant cells bordering the internal elastic lamina and significantly reduced lumen.

The patient was diagnosed with GCA and commenced on 80 mg of prednisone daily with regular testing of his CRP. Over the next 6 months his visual acuity improved to 6/36 in the left eye. Ophthalmoscopy at this time revealed large areas of atrophic chorioretinal scars in the left eye.

## 3. Discussion

GCA is the most common type of vasculitis affecting people aged over 50 years. It rarely affects people younger than 50 years of age, and the incidence rises markedly with increasing age, peaking in the eighth decade of life. The highest frequencies have been reported from Scandinavian countries and populations of predominantly Scandinavian descent, such as Minnesota, USA and the UK. Incidence rates in southern Europe and Israel are intermediate. GCA seems to be infrequent in people of African, Asian, Hispanic, and Arab descent, but data are sparse [2].

It is widely believed among ophthalmologists that patients with GCA always present with systemic symptoms and signs (fever, headache, and jaw claudication) and that the absence of such symptoms and signs rules out GCA. This impression has sometimes, sadly, caused misdiagnosis and consequent blindness. Development of visual symptoms as the first or the only sign of GCA was first reported in 1952 [3].

With regards to pathophysiology, when the posterior ciliary arteries of the eye are affected, an optic nerve infarction with subsequent Anterior Ischemic Optic Neuropathy (AION) is usually caused [4]. AION with visual loss and visual field loss occurs in 80%–90% of the cases with ocular involvement. Occlusion of the posterior ciliary arteries may, in addition to arteritic AION, cause patches of choroidal infarcts that appear as peripheral chorioretinal degenerative lesions 2 to 3 weeks later. They are usually located in the midperipheral region of the fundus and frequently are triangular in shape, with their base toward the equator and apex toward the posterior pole [5].

Early diagnosis of GCA and early and aggressive treatment are the keys to preventing blindness in one or both eyes. Thus, any patient over the age of 55 years who presents with a history of amaurosis fugax, diplopia, or visual loss and has clinical findings of arteritic anterior ischemic optic neuropathy, central retinal artery occlusion, cilioretinal artery occlusion, or posterior ischemic optic neuropathy should be suspected of having GCA and have erythrocyte sedimentation rate and CRP level evaluations done on an emergency basis, regardless of having systemic symptoms of GCA. If, based on this information, there is a high index of suspicion, then the patient should be started immediately on high doses of systemic corticosteroids, and a temporal artery biopsy should be performed to confirm or rule out GCA.

The most important factor is how early the diagnosis of GCA is established and the treatment started; the sooner GCA is diagnosed and managed correctly, the lower the incidence of visual loss.

Summarizing, we report an unusual presentation of choroidal infarction associated with GCA and emphasize the need of thorough systemic evaluation in patients with choroidal infarction.
